# Supplementary material for: Inhibition of PCSK9 Attenuates Liver Endothelial Cell Activation Induced by Colorectal Cancer Stem Cells During Liver Metastasis
Source: Cancers (Basel). 2025 Jun 13;17(12):1977. doi: 10.3390/cancers17121977 (PMC12190733; doi:10.3390/cancers17121977)
Supplement: Supplementary file 1 [file cancers-17-01977-s001.zip › cancers-3644648-supplementary.pdf]

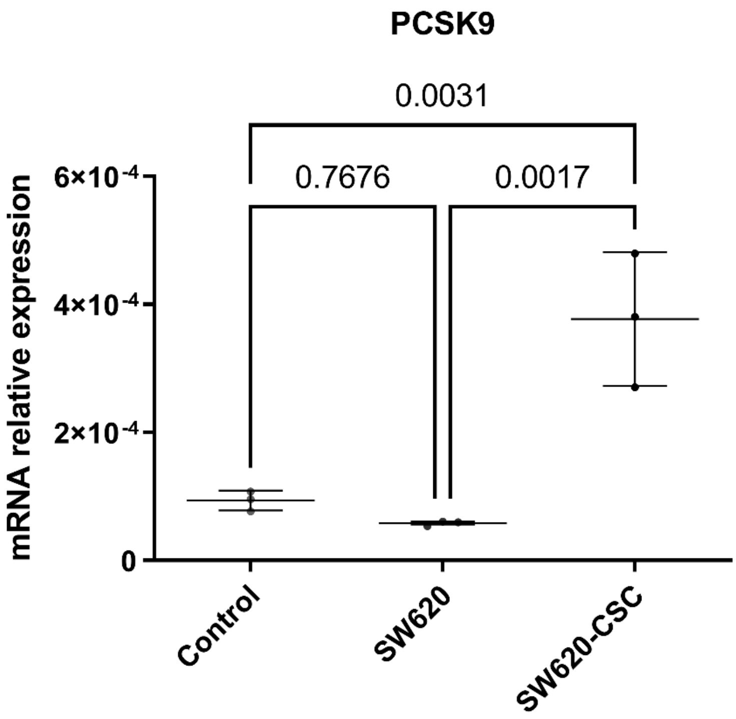

Supplementary Figure S1: Preliminary study of PCSK9 expression in primary human LSEC. Activation with conditioned mdia from SW620-CSC seems to significantly increase PCSK9 expression. Two-way ANOVA (n=3), Tukey's post hoc test: ^{ns} p>0.05; *** p<0,001.

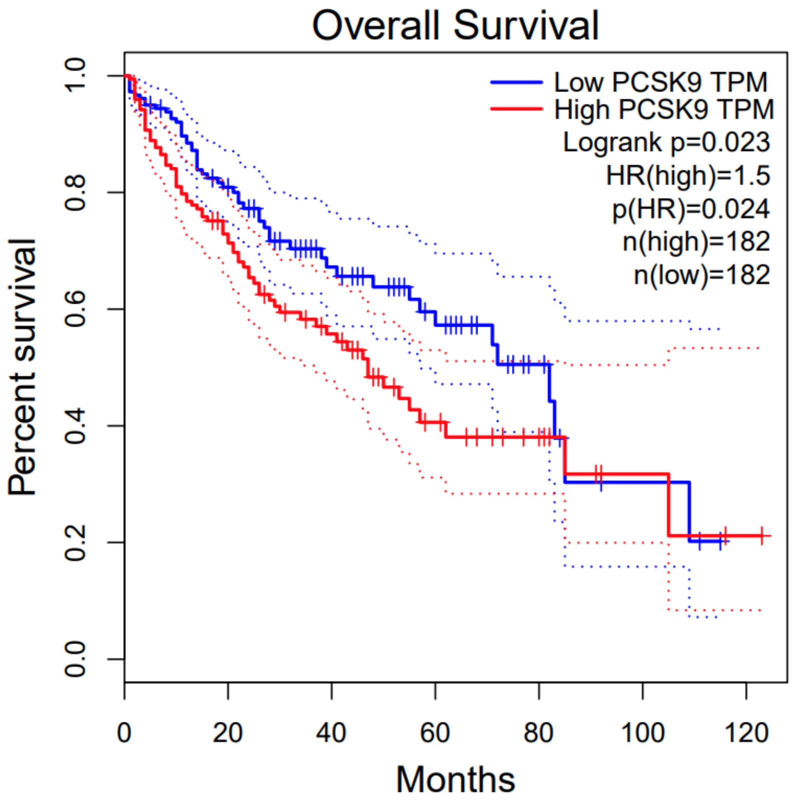

Supplementary Figure S2: Overall survival of liver hepatocarcinoma patients depending on PCSK9 expression. A higher expression of PCSK9 results in a significantly lower survival rate in Liver hepatocarcinoma.
